# Investigating the Epigenetic Landscape of Major Depressive Disorder: A Genome-Wide Meta-Analysis of DNA Methylation Data, Including New Insights into Stochastic Epigenetic Mutations and Epivariations

**DOI:** 10.3390/biomedicines12102181

**Published:** 2024-09-25

**Authors:** Giulia Nicole Baldrighi, Rebecca Cavagnola, Luciano Calzari, Davide Sacco, Lucy Costantino, Fulvio Ferrara, Davide Gentilini

**Affiliations:** 1 Department of Brain and Behavioral Sciences, Università di Pavia, 27100 Pavia, Italy; giulianicole.baldrighi01@universitadipavia.it (G.N.B.); rebecca.cavagnola01@universitadipavia.it (R.C.); davide.sacco02@universitadipavia.it (D.S.); 2 Bioinformatics and Statistical Genomics Unit, Istituto Auxologico Italiano IRCCS, 20095 Cusano Milanino, Italy; l.calzari@auxologico.it; 3 Medical Genetics Laboratory, Centro Diagnostico Italiano, 20147 Milan, Italy; lucy.costantino@cdi.it; 4 Integrated Laboratory Medicine Services, Centro Diagnostico Italiano, 20147 Milan, Italy; fulvio.ferrara@cdi.it

**Keywords:** MDD, epigenetic drift, rare epivariations, epigenetics

## Abstract

**Background/Objectives:** Major depressive disorder (MDD) is a mental health condition that can severely impact patients’ social lives, leading to withdrawal and difficulty in maintaining relationships. Environmental factors such as trauma and stress can worsen MDD by interacting with genetic predispositions. Epigenetics, which examines changes in gene expression influenced by the environment, may help identify patterns linked to depression. This study aimed to explore the epigenetic mechanisms behind MDD by analysing six public datasets (n = 1125 MDD cases, 398 controls in blood; n = 95 MDD cases, 96 controls in brain tissues) from the Gene Expression Omnibus. **Methods:** As an innovative approach, two meta-analyses of DNA methylation patterns were conducted alongside an investigation of stochastic epigenetic mutations (SEMs), epigenetic age acceleration, and rare epivariations. **Results:** While no significant global methylation differences were observed between MDD cases and controls, hypomethylation near the SHF gene (brain-specific probe cg25801113) was consistently found in MDD cases. SEMs revealed a gene-level burden in MDD, though epigenetic age acceleration was not central to the disorder. Additionally, 51 rare epivariations were identified in blood tissue and 1 in brain tissue linked to MDD. **Conclusions:** The study emphasises the potential role of rare epivariations in MDD’s epigenetic regulation but calls for further research with larger, more diverse cohorts to confirm these findings.

## 1. Introduction

Major depressive disorder (MDD) is a medical condition placed within a broader category of depressive disorders, acknowledging that different disorders within this spectrum share overlapping features; this is a result of efforts to organise mental health disorders more consistently based on shared biological and clinical characteristics [1]. Currently, MDD is classified in the Diagnostic and Statistical Manual of Mental Disorders, Fifth Edition (DSM-5) as a mental disorder characterised by a persistent and pervasive low mood, loss of interest or pleasure in activities, and a variety of cognitive and physical symptoms that interfere with daily functioning [2]. The DSM-5, a key classification system in the U.S. for mental disorders, aligns with the International Classification of Diseases (ICD), particularly with efforts made to harmonise criteria for international consistency, as seen in both DSM-5 and ICD-11 developments [1]. The diagnosis of MDD according to the DSM-5 involves a set of explicit criteria, including the presence of at least five of the following symptoms over a two-week period, with at least one being either a depressed mood or loss of interest or pleasure, i.e., (1) low mood nearly every day; (2) markedly diminished interest or pleasure in almost all activities; (3) significant weight loss or gain, or changes in appetite; (4) insomnia or hypersomnia; (5) psychomotor agitation or retardation; (6) frequent fatigue or loss of energy; (7) feelings of worthlessness or excessive guilt; (8) diminished ability to think or concentrate, or indecisiveness; (9) recurrent thoughts of death, suicidal ideation, or a suicide attempt. The manual was updated to integrate findings from neuroscience, genetics, and clinical studies, moving away from purely descriptive categorisations.

As for the genetic nature of MDD, several genes, including transporters, neurotransmitters, and neurotrophins, may predispose individuals to MDD. However, significant gaps remain in our understanding of MDD, such as the precise regulatory mechanisms governing the clinical spectrum, the interaction between genetic and environmental factors, and the reasons for variability in treatment responses.

Exploring the field of epigenetics may offer promising insights into the complex aetiology of MDD and potential advancements in therapeutic strategies, as evidenced by recent studies investigating DNA methylation changes in both brain and blood tissues [3,4,5,6,7,8].

Studies exploring genome-wide DNA methylation in MDD have identified significant associations with the disorder [9,10]. However, excluding the study by Jovanova et al. (2018) [11] conducted on whole blood that involved a large and heterogeneous sample, the other studies, particularly those analysing brain tissue DNA, are characterised by a smaller sample size. This limitation, along with the lack of replication across studies and a limited understanding of the functional implications of the identified associations, raises important questions about the generalisability and biological relevance of these findings. This highlights the need for further research with improved statistical power to clarify the role of epigenetic modifications in MDD. Previous studies have also investigated other aspects of epigenetic regulation in MDD patients, including differences in age acceleration. However, also in this case there is a lack of uniform agreement in the results, with some studies observing no significant differences in age acceleration [12] and others highlighting differences between cases and controls [13,14,15].

Furthermore, previous studies have largely neglected other intriguing aspects of epigenetic regulation such as epigenetic drift and rare epigenetic variations (epivariations), two underexplored areas of DNA methylation variability. These aspects are increasingly recognized as fundamental for describing the epigenetic landscape involved in phenotype modulation and have been described in various physiological and pathological conditions. Epigenetic drift refers to the gradual accumulation of stochastic epigenetic changes over an individual’s lifespan. The gradual changes in the epigenome can lead to dysregulation of key genes and pathways, potentially increasing susceptibility to psychiatric illnesses and influencing the course of the disease [16,17]. This study aims to investigate the role of DNA methylation and other epigenetic modifications potentially acting during MDD, focusing on both brain and blood tissue. We hypothesised that distinct patterns of epigenetic variability, including epigenetic drift and rare epigenetic variations, contribute to the onset and progression of MDD, offering novel insights into its complex aetiology and potential therapeutic targets. Our research reflects the lack of a starting point to provide consistency to epigenetic areas to be explored to characterise complex diseases, where genetics interacts with the environment.

## 2. Materials and Methods

### 2.1. Selection of Datasets

To carry out this project, a database search was performed for studies up until January 2024 using the keywords major depressive disorder, methylation, and case–control, in the Gene Expression Omnibus (GEO) data repository and the EWAS Data Hub platform. To be included, studies had to be case–control studies, reporting data using the Illumina HumanMethylation450 or Infinium MethylationEPIC platforms, in blood tissue or in brain districts. Only public data were included.

### 2.2. Methylation Quality Control Data Preprocessing and Differential Methylation Analysis

Each dataset was analysed separately with the same procedure. First, data quality control was performed at both the sample and probe levels. This involved identifying technical and biological biases, errors in sampling, and other confounders. Probes with high or low fluorescence and those with insignificant β values compared to the background were discarded, along with probes that hybridised at the sex chromosome level and sites containing SNPs and known natural C/T polymorphisms. Conversion efficiency index was checked for each sample, and samples with a low index were excluded. Outliers and samples with non-bimodal methylation levels or less than 99.5% of CpG sites determined successfully were also excluded. Putative homozygous deletion regions were identified and removed. Differential methylation analyses were conducted using *Champ* [18] and *limma* [19] packages. Before proceeding in the differential analysis, data underwent singular value decomposition (SVD) [18], applied with the *prcomp* function, for considering batch effects emerging from a sample’s Illumina Sentrix ID and its position during the array experiment. The SVD method in differential analysis exploration serves to reduce data dimensionality, helping to identify key patterns and eliminate noise, which improves the detection of significant differences between groups due to experimental conditions. If present, such errors were adjusted using the *ComBat* method [20], which is a gold standard method used with array data for non-biological variability across different batches while preserving the biological signals of interest.

Principal component analysis (PCA) was employed to reduce the complexity of cellular components estimates. This solution enabled us to utilise the PCs as covariates in comparative models between the two groups, effectively sidestepping collinearity issues. The estimation of cellular components was conducted using the *epidish* package [21], for deconvoluting heterogeneous tissue samples by estimating the proportions of different cell types in DNA methylation data. It helps to correct for cellular heterogeneity in epigenetic studies, improving the accuracy of differential methylation analysis.

After normalising to address batch effects, differential methylation analyses were performed at both site and genomic region levels using hierarchical linear models implemented in the *limma* package, adjusting for potential confounders such as age, sex, batch effect, and PCs. False discovery rate (FDR) was adopted to address the problem of multiple tests. The pipeline was entirely performed in R Studio version 4.4.1 (14 June 2024).

### 2.3. Age Acceleration Analysis

Epigenetic ageing was assessed using the GrimAge clock, which calculates age acceleration by integrating DNA methylation-based surrogates for various proteins, biological markers, and self-reported smoking history [22]. These measures were derived from DNA methylation data using the *dnaMethyAge* package [23] with the PCGrimage clock. To examine the association between the GrimAge clock and MS, a general linear model was employed under the null hypothesis of no effect of MS on the mean difference between epigenetic age and chronological age, adjusting for sex and the principal components of cellular compositions.

### 2.4. Epigenetic Drift and Stochastic Epigenetic Mutations Analysis (SEMs)

Stochastic epigenetic mutations (SEMs) refer to infrequent, random, and inconsistent alterations in DNA methylation. These changes are determined through the examination of DNA methylation data, as outlined by Yan et al. [24]. Upon identification of extreme outliers in CpG probe methylation levels within a population, outliers are determined as methylation levels surpassing three times the interquartile range (IQR), as described in the following formula:

Q1 − (3 × IQR) and Q3 + (3 × IQR)(1)

Additionally, following the approach by Yan et al. (2020) [24], SEMs were utilised to evaluate epigenetic drift in each subject, generating two epimutation load (EML) scores. These scores aim to quantify the overall burden of SEM counts across the entire genome (Global-EML) and at the gene level (Gene-EML). To explore the relationship between Global-EML and MDD, a logistic regression model was employed, incorporating the same covariates used in the EWAS step. The burden of SEMs was log-transformed and compared between cases and controls. The sequence kernel association test (SKAT) was adopted to explore associations between MDD and Gene-EML (i.e., gene-specific epigenetic drift scores). SEM calls at each methylation probe were treated as the variants of interest, following the approach outlined by Chen et al. (2022) [25]. The RVTEST, initially designed for mapping a contiguous set of rare variants to a specific trait, was extended to accommodate other measures like copy number variants (CNV) and methylation counts. This extension operated under the assumption that a cluster of variations in adjacent sites could be relevant to the trait. The SKAT enhanced the statistical power to identify associations with the burden of SEMs by considering the joint effect of multiple rare epigenetic variants. The analyses were conducted on both blood and brain tissue, and the results obtained from each study, depending on the tissue, were aggregated using Fisher’s method [26].

### 2.5. Epigenetic Variation Analysis

The identification of epigenetic variations followed a validated approach outlined by Gentilini et al. (2023) [27,28]. This method involved scrutinising genomic regions with a significant enrichment of SEMs. A sliding window method of a predefined size was used on the annotated genome to assess significant SEM enrichment regions, employing a hypergeometric distribution. The algorithm systematically tested each window by sliding it (one site at a time), generating *p*-values associated with each window. The concept of epivariations encompasses regions with abnormal methylation patterns and a notable increase in epimutations, as detailed by Garg et al. and Gentilini et al. [27,29]. These variations in DNA methylation states can arise from various origins. Primary epivariations result from random errors during the establishment or maintenance of the epigenome, especially during cellular differentiation. These errors are sporadic and not necessarily linked to changes in the DNA sequence. Additionally, genetic factors like CNVs, CGG repeats, and single nucleotide variations (SNVs) can influence the epigenome, leading to epivariations. Understanding these sources is key to uncovering the underlying molecular mechanisms. To identify epivariations, we used a method validated by Gentilini et al. (2018) [27], involving the detection of over-representation of epimutated probes for each subject through a sliding window approach. This method assessed the build-up of epimutations using the hypergeometric cumulative function, with each epivariation represented by a *p*-value indicating the likelihood of epimutation enrichment in the specified genomic region.

### 2.6. Meta-Analysis

The meta-analysis was performed to combine results from differential methylation analyses for both tissues. This synthesis was conducted separately for blood and brain samples to ensure comprehensive analysis. In the context of brain differential methylation analysis, separate investigations were carried out for different brain areas. However, the individual findings from these separate studies were later combined and summarised together in a meta-analysis. The meta-analyses aimed to provide a more comprehensive and integrated understanding of the overall differential methylation patterns across various brain regions, offering valuable insights into the epigenetic differences that may be associated with specific brain functions or conditions. The software METAL was used, which is a specific tool to perform meta-analyses at genome-wide and epigenome-wide levels [30]. Moreover, the same approach was applied to gather information on age acceleration analysis and epigenetic drift for both tissues. A mixed-effects model was applied, in which the hypothesis is that the observed differences could be due to differences between studies. The goal was to obtain a summary estimate for the effect size. This analysis was conducted through the *metafor*, which provided, in addition to pooled estimates, statistics regarding heterogeneity such as the Q statistic and the I^2^ statistic [31].

### 2.7. Gene Set Enrichment Analysis and Gene Prioritisation Analysis

To perform the over-representation analysis (ORA) on epivariations, a prioritisation was firstly performed to highlight the most interesting genes associated with epivariations. Priority was given to genes detected in three cases or more but not in controls across both brain and blood tissues. Subsequently, we refined our gene selection by excluding those already identified in a previous study involving 23,116 subjects from the general population [29]. The filtered genes’ list underwent additional investigation using the Genotype-Tissue Expression (GTEx) project [32] to identify genes expressed in the brain. Additionally, the VarElect tool [33] was employed to spotlight genes already recognised for their involvement in MDD.

## 3. Results

### 3.1. Datasets and Differential Methylation Meta-Analyses

**Figure 1:**
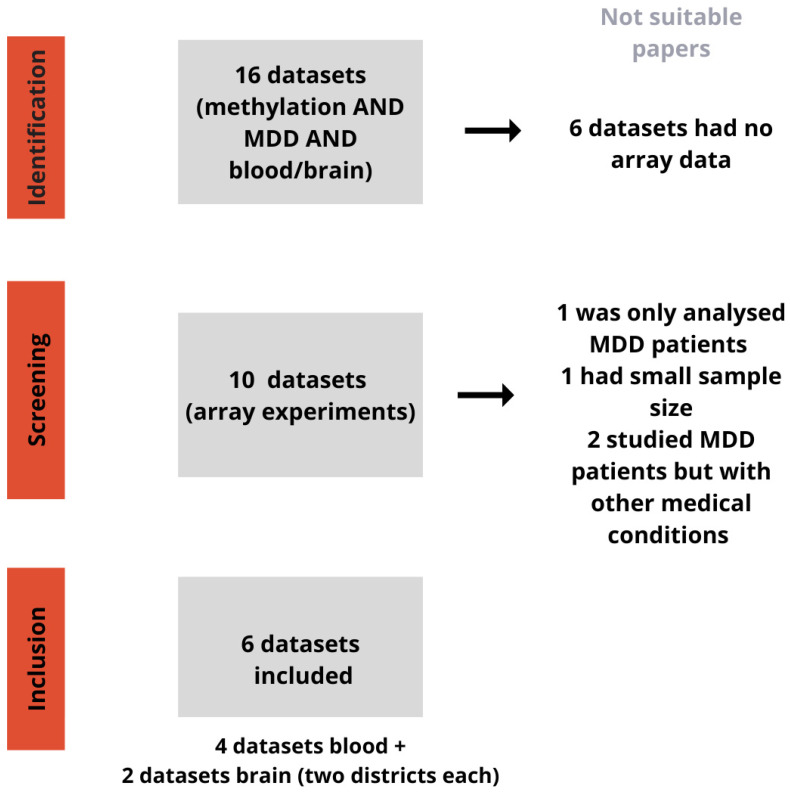
Workflow for dataset selection. This picture shows the workflow for identifying datasets related to methylation and MDD in blood and brain samples. During the identification step, 6 out of 16 datasets were discarded due to lack of array data. From the remaining 10 datasets, 4 were excluded for various reasons: one only analysed MDD patients, one had a small sample size, and two studied MDD patients with other medical conditions. Eventually, 6 datasets were included for analysis: 4 from blood samples and 2 from brain samples (with two brain districts each).

We included only studies with a sample size greater than 20. After the application of the previous criteria, datasets from six studies were eligible and included in the analyses (GSE113725, GSE201287, GSE125105, and GSE198904 were obtained from blood tissues while GSE88890 and GSE41826 were obtained from brain tissues). The studies referred to brain tissue containing information on different areas of the brain. Specifically, GSE41826 was divided into glia and neurons and GSE88890 into area BA11, located in the orbitofrontal cortex, and BA25, a region of the cerebral cortex. The information about the selection workflow and the included datasets, with respective publications [3,4,5,6,7,8], is presented in Figure 1 and Table 1. The analysis was performed on four datasets for blood, for a total of 1125 MDD cases and 398 controls, and two datasets for brain tissues, involving two areas each, for a total of 95 MDD cases and 96 controls.

**Figure 2:**
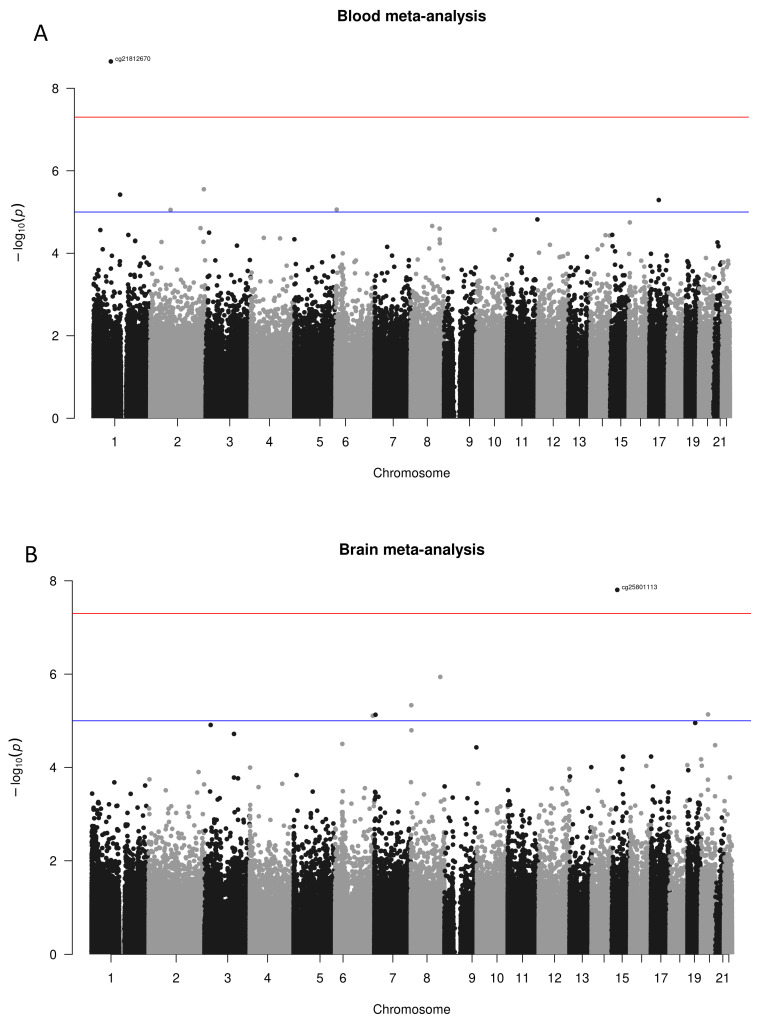
Manhattan plots of meta-analysed differential methylation analyses, divided by results observed in blood tissue (**A**) and brain tissue (**B**). In both graphs, the x-axis represents the chromosomes where the probes are located. The y-axis represents the negative base-10 logarithm of the association *p*-value, with a red horizontal line indicating the threshold for significant unadjusted *p*-value (suggestive significance threshold shown with the blue line at 10^−5^). Few reached the suggestive threshold, while only two probes cg21812670 in blood and cg2580113 in the brain went even beyond the 10^−7^ (red line). Note that the only probe still significant (FDR < 0.05) after the multiple testing correction was the cg25801113.

The exploratory analyses, conducted separately in each dataset for both blood and brain tissues, aimed to identify overarching differences in methylation between cases and controls, as well as detect batch effects and hidden confounders. These analyses did not reveal any significant distinctions between the groups analysed. After normalising to account for batch effects, differential methylation analysis was performed at the probe level, considering potential confounding factors detailed in the Methodology section. The results from these analyses were then systematically integrated using a meta-analytic approach. The findings from the meta-analyses are depicted in Manhattan plots for blood and brain tissues, as illustrated in Figure 2 (Panels A and B, respectively), where unadjusted *p*-values distributions are shown. After applying the FDR multiple testing correction in the meta-analysis of blood tissue, no probes showed significant differential methylation (FDR < 0.05). In contrast, in the meta-analysis of brain tissue, one specific probe (cg25801113) consistently exhibited significant differential methylation across multiple studies, even after the multiple testing correction. This significant probe is in the region of interest associated with the SHF gene. The comprehensive meta-analysis results for blood and brain tissues are respectively shown in Supplementary Tables S1 and S2.

ORA was conducted for both blood and brain tissues using nominal significance to consolidate significant findings and identify biochemical pathways potentially involved in depression. This approach enables the exploration of biological trends and functional pathways that may not reach statistical significance after multiple testing corrections, enhancing sensitivity to uncover subtle but meaningful associations. ORA uncovered enrichment in various gene ontology biological processes, particularly highlighting neurogenesis and positive regulation of gene expression. Moreover, analysis at the KEGG pathway level indicated significant enrichment in neurotrophins signalling and the Hippo signalling pathway. Detailed findings from ORA performed on whole blood tissue are available in Supplementary Table S3.

Regarding brain tissues, the most interesting results of ORA considering biological processes were locomotion, neurogenesis, and the regulation of cell differentiation. The complete list of results obtained from ORA in the brain is shown in Supplementary Table S4.

### 3.2. Age Acceleration

**Figure 3:**
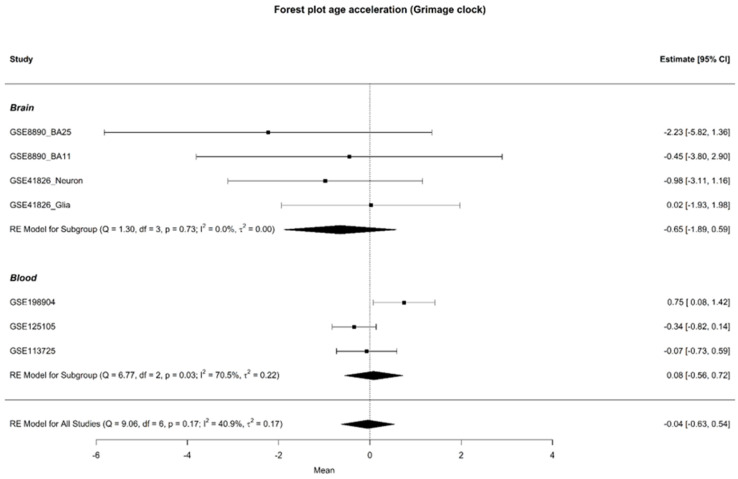
Forest plot of age acceleration in brain and blood samples, with individual study results on the y-axis, excluding study GSE201287. The forest plot displays the observed outcomes as mean differences on the x-axis and the 95% confidence intervals resulting from the analysis of the association between age acceleration Grim and depression, based on a linear regression model. The vertical line highlights the area of no effect (mean difference = 0), with the size of each square proportional to the study’s influence on the overall estimate [3,4,5,6,7,8].

Epigenetic age was assessed in all participants with available chronological age data, with the exception of those in study GSE201287, which did not provide chronological age information. Age acceleration was evaluated using various epigenetic clocks across both brain and blood samples, but no differences were identified between cases and controls. The results from the GrimAge clock are reported here, as it is considered a second-generation epigenetic clock, offering advanced insights into age-related changes. However, no significant difference in age acceleration was observed between the groups, with mean differences of −0.65 [95% CI: −1.89; 0.59] for brain samples and 0.08 [95% CI: −0.56; 0.72] for blood tissue. A forest plot illustrating these findings is provided in Figure 3.

### 3.3. SEM Analysis and Epimutation Load

**Figure 4:**
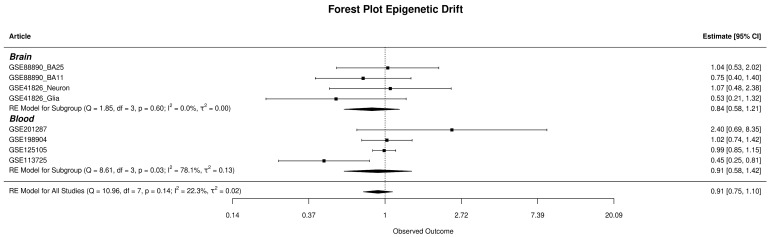
The forest plot displays the observed outcomes as odds ratios on the x-axis and the 95% confidence intervals resulting from the analysis of the association between epigenetic drift and depression, based on a logistic regression model. The vertical line highlights the area of no effect (OR = 1), with the size of each square proportional to the study’s influence on the overall estimate [3,4,5,6,7,8].

The examination of epigenetic drift encompassed both global and gene-specific levels, utilising two distinct metrics: Global-EML, which spans the entire genome, and Gene-EML, focusing on individual genes. These metrics allow for the assessment of cumulative SEM counts across the genome and at the gene level, offering a comprehensive evaluation of the overall SEM burden for everyone. The Global-EML scores were log-transformed and then compared between groups within each study using a multiple regression model, which accounted for crucial covariates including gender, age, and cellular components. These analyses were conducted separately for each study, and the results were later integrated through meta-analysis to achieve a pooled estimation (Figure 4). After combining the results, the analysis found no significant effect of the epimutation load on the risk of developing MDD in either whole blood (OR = 0.91, CI = [0.58–1.42]) or brain tissues (OR = 0.84, CI = [0.58–1.21]). A comprehensive display of the meta-analysis results regarding Global-EML is presented in Figure 3.

Gene-EML scores were analysed using a sequence kernel association test (SKAT) within each dataset, to evaluate differences in epigenetic drift at the gene level between cases and controls. In both brain and blood tissues, Fisher’s method was then used to combine results from the SKAT tests and detect genes displaying significant differences in epigenetic drift between cases and controls across all studies. The comprehensive results for Gene-EML are available in Supplementary Table S5.

ORA was performed to uncover biological elements that could be implicated or influenced by the observed epigenetic drift. The investigation into gene ontology (GO) terms and pathways in blood uncovered significant associations with MDD. Noteworthy findings included the involvement of the Wnt signalling pathway and cell–cell signalling by Wnt in biological processes, as well as KEGG pathways linked to mTOR signalling, neurodegeneration, and inflammation. In brain tissue, significant associations were found with KEGG pathways such as the adipocytokine signalling pathway, glycerophospholipid metabolism, and regulation of lipolysis in adipocytes, alongside biological processes related to regulation of neurotrophins production, nerve growth factor production, and positive regulation of response to extracellular stimuli. Detailed results are available in Supplementary Tables S6 and S7.

### 3.4. Epigenetic Variation Analysis Results

We broadened our investigation to highlight genes potentially implicated in MDD within the framework of epivariations. Distinct regions with significant enrichment in SEMs were prioritised both in blood and brain tissues. The selection was performed considering genes’ regions from all studies, where epivariations did not appear in controls and appeared in at least three or more cases. Based on the criteria established, 192 genes were identified in whole blood and 1 in the brain.

**Figure 5:**
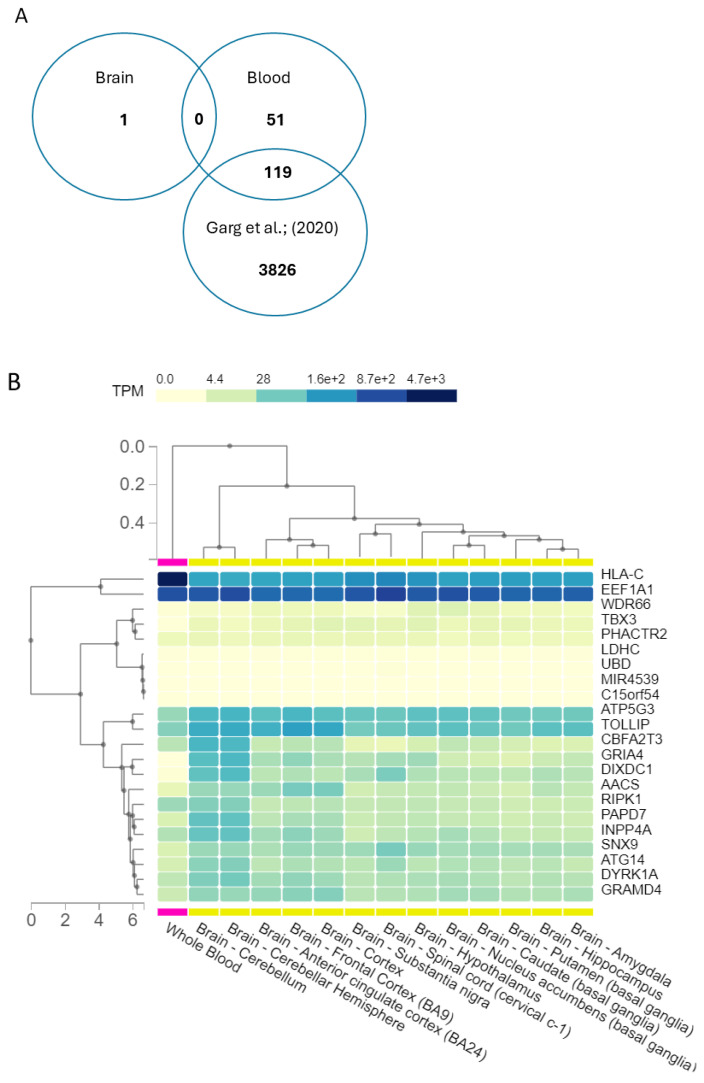
(**A**) Venn diagram in describing the intersection of genes lists. From epivariations, we identified 51 genes from blood tissues and 1 gene emerged from the brain. An intersection was performed with the list of genes involved in epivariations in blood, the list with epivariated genes in brain, and the list with epivariations provided by Garg et al. (2020) [29]. (**B**) Expressions of the epivariated genes found in brain and blood. The expressions of the epivariated genes (present in 0 controls and in more than 3 cases) were analysed with GTEx. Expression is quantified as transcripts per million (TPM), which is the ratio of number of transcripts per gene over the total number of transcripts in the sample multiplied by 106.

Subsequently, we refined our gene prioritisation by excluding those already identified in a previous study by Garg and colleagues (2020) [29] involving more than 23,000 subjects from the general population, as described in the Methods section. The Venn diagram describes the filtering approach used. This analysis identified 51 genes from blood tissues and 1 gene emerged from the brain. These genes underwent further investigation using GTEx to explore their expression profiles in brain and blood tissues, to see whether expressions in the two tissues might appear similar. Then, a focused examination was conducted on the genes with the highest expression levels in brain tissue using GTEx, and the results are illustrated in Figure 5B. The expression was quantified as transcripts per million (TPM), which is a method used to normalise gene expression data to account for differences in gene length and sequencing depth.

## 4. Discussion

Exploring the epigenetic landscape of MDD led us to comprehensively evaluate one of the plausible solutions where the interplay between genetic and molecular factors contributing to MDD may arise. Implementing a meta-analytical approach for whole blood and brain tissues, the aim was to pool results of methylation patterns in the MDD context from different studies to increase the power and explore heterogeneity among studies. Alongside finding methylation similarities from different studies already investigating MDD epigenetics, our focus was on the age acceleration and rare epigenetic variations. The analysis involved 1125 MDD cases and 398 controls for whole blood and 95 MDD cases and 96 controls for brain tissues.

In the differential meta-analysis of whole blood, no probes showed significant differential methylation between cases and controls after FDR correction. This rarefied signal aligns with a recent study involving nine population cohorts with over 10,000 subjects, which identified only three significantly associated probes [11]. Although not confirming the same probe, because they were not included in the datasets we selected from publicly available data, we found that MDD phenotype has no particular pattern highlighting the differential methylation when considering the epigenetic-wide association study, considering whole blood methylome. Given the large sample size and statistical power of the Jovanova et al. (2018) study [11], it is not surprising that no significant genome-wide results emerged in our analysis. The lack of clear epigenetic signatures for MDD may reflect the disorder’s heterogeneity, suggesting that MDD may involve a complex and varied set of underlying mechanisms not explainable by epigenetics alone. Conversely, in the analysis of the brain tissue districts, a particular probe (cg25801113) consistently demonstrated significant hypomethylation across multiple studies, even after FDR correction. This finding highlights the substantial hypomethylation of the probe, situated within the region of interest linked to the SHF gene, particularly interesting in the depression context since its neurobiological implications have already been studied in Alzheimer’s disease (AD). The SHF protein is expressed in the brain, including the hippocampal formation, amygdala, basal ganglia, midbrain, spinal cord, cerebral cortex, cerebellum, hypothalamus, and choroid plexus, mostly linked to emotional regulation and neurobiological processes [34]. An integrated analysis of human genetic association and the mouse transcriptome suggests that the SHF gene is a novel susceptible gene for amyloid-beta (Aβ) [34]. Hypomethylation generally correlates with increased gene expression. The consistent hypomethylation of cg25801113 suggests a regulatory role of epigenetic mechanisms over the SHF gene in brain tissue. This could imply altered expression of SHF in individuals with depression, potentially influencing neurological pathways relevant to emotional regulation.

The ORA conducted on probes reported a suggestive significance, i.e., *p*-value < 10^−5^ [35], both for blood and brain tissues. ORA in whole blood highlighted several enriched biological processes (BP) terms, among which the most interesting were neurogenesis and positive regulation of gene expression, while KEGG pathways showed a significant enrichment in neurotrophin signalling and Hippo signalling. The association of the Hippo signalling pathway with bipolar disorder (BD) and its positive transcriptional regulation might characterise its involvement. Perturbations in this pathway may result in abnormalities in neural progenitor cell maintenance and asymmetric division, crucial for generating cortical neurons, potentially influencing mood regulation and the onset of depressive symptoms [36,37]. Moreover, the observed enrichment in neurogenesis and positive regulation of gene expression holds significant relevance to depression [38,39]. These findings align with the recent study by Jovanova et al. [11], which reached similar conclusions. As for brain tissue, the most significant pathways refer to locomotion and neurogenesis. The involvement of all these processes in depression may offer potential targets for intervention and further our understanding of the molecular mechanisms underlying the condition.

Epigenetic drift and SEMs were investigated, both globally and at the gene level. While the Global-EML did not indicate an elevated drift, the Gene-EML, which depicted a notably distinct SEM burden at the gene level between cases and controls, revealed a catalogue of 50 genes in the blood and 10 genes in the brain. The findings revealed a significant accumulation of mutations in the methylation patterns of specific genes. Notably, in blood, the ORA highlighted several significantly enriched pathways, including the mTOR signalling pathway (FDR = 0.01), neurodegeneration-multiple diseases (FDR = 0.006), and carbohydrate digestion and absorption (FDR = 0.004). The mTOR pathway, a key regulator of homeostasis, impacts protein synthesis, autophagy, and metabolism. Dysregulation of mTOR signalling has been directly associated with MDD, and pharmacological modulation of this pathway shows promise as a treatment for depression [40]. The PI3K/Akt/mTOR signalling pathway has been associated with neurodegenerative diseases and is involved in regulating a wide range of upstream molecules, including those related to inflammation and oxidative stress [41]. In the context of depression, the interplay between stress, inflammation, and immune dysregulation has been shown to contribute to atrophy and loss of neurons and glia, which are central to the pathophysiology of the disorder [42]. In line with recent studies, the association between sugar malabsorption and depressive symptoms in adult women has been reported [43]. Incompletely absorbed carbohydrates may form nonabsorbable complexes with tryptophan, decreasing its availability and leading to serotonin depletion, which could contribute to depression [44]. Hence, they can be the target of an ample range of combined treatments, each operating via diverse mechanisms. In the brain districts, while no significant pathway was found from ORA, significant and interesting biological processes linked to the relationship between MDD and digestive system emerged, e.g., positive regulation of appetite, positive regulation of response to nutrient levels. Moreover, the pathway of regulation of neurotrophin production emerged.

An additional level of analysis focused on rare epivariations, which are regions displaying abnormal methylation patterns and are characterised by a notable increase in epimutations; rare epivariations have been linked to various neurological conditions such as ALS [45] and autism spectrum disorder [17], but their potential role in MDD has yet to be fully explored. This analysis identified 959 distinct genes with epivariations found exclusively in blood tissue from MDD cases, and a separate set of 180 unique genes with epivariations observed solely in brain tissue from MDD cases. These epivariations were specific to MDD and were not present in control subjects. To prioritise the most compelling findings, we selected genes with epivariations in at least three cases and absent in controls for both tissues. To further refine our list, we excluded genes that were identified in a study by Garg et al. [29], which involved over 23,000 subjects from the general population. This approach yielded a list of epivariations unique to MDD cases. This selection highlighted 1 gene in the brain and 51 genes in blood. The identified genes were initially prioritised by reviewing those previously studied in the context of MDD. To further refine this gene list, we employed a method similar to the approach used in exome sequencing analysis with the *VarElect* tool. This approach allowed us to focus on genes with a higher likelihood of being relevant by filtering out less significant findings and homing in on those with substantial epivariations specifically associated with MDD. Particularly interesting were the two genes directly linked to MDD, i.e., GRIA4, the protein-coding gene for subunit 4 of the AMPA glutamate receptor involved in glutamate signalling and neuroplasticity [46], which may be implicated in psychiatric disorders, together with the GRIA1 and GRIA2 genes [47]. The other relevant gene was GAS5, an lncRNA gene that was investigated for being a biomarker for type 2 diabetes, which might be also involved in MDD progression [48] through mRNA regulation. In mice models, GAS5 was studied for depression-like behaviors, and its downregulation was found to alleviate hippocampal neuronal damage [49]. This list of unique epivariations, was subsequently investigated in order to obtain their expression profiles in blood and brain tissues. Among genes not directly associated to MDD, there were genes involved in immune system reactions and autophagy (CD4, HLA-C, ATG14), proteins implicated in the phosphorylation process and with the role of kinases (DYRK1A, RIPK1), and transferase (CAT1, GALNT2). GALNT2 appeared to be upregulated in the striatum but decreased in the hippocampus of MDD patients, suggesting it may have different functions in different brain regions in MDD progression [50].

While this study identifies rare epigenetic variations unique to MDD cases, it faces several limitations. First, the datasets used in the meta-analyses have unidentifiable heterogeneity due to varying sampling techniques. Although the meta-analytical approach increased sample size, its robustness cannot match Jovanova et al. (2018) [11], who used a single-study design to reduce heterogeneity. Additionally, confounding factors such as drug use, lifestyle, and comorbidities could not be fully addressed due to missing information in the original studies. Finally, our study cannot explore the interaction between genetics and epigenetics, focusing only on exploring the existence of rare epivariations related to neuronal dysfunction in MDD patients.

## 5. Conclusions

In conclusion, this study explored the epigenetic regulation of MDD, focusing on rare epivariations and stochastic epigenetic mutations, areas previously underexamined. The research offers new insights into the molecular mechanisms of MDD, highlighting the potential role of epigenetic modifications in the disorder’s onset and progression. Despite the strengths of a meta-analytical approach, limitations include variability in sample sizes and genetic diversity, which could underestimate heterogeneity and complicate the identification of consistent epigenetic markers, particularly in blood and brain tissues. The lack of detailed phenotypic data further restricts the depth of analysis. While no specific epigenetic signature, accelerated ageing, or significant epigenetic drift was found, the study did reveal potentially important rare epivariations in MDD. The discovery of rare epivariations holds significant promise for future research, emphasising their potential as novel diagnostic markers or therapeutic targets, thereby reinforcing their importance in advancing personalised medicine.

## Figures and Tables

**Table 1:** Dataset included in the analysis. The table describes the selected studies downloaded from the GEO database with main information about GSE code, tissue of methylated data extraction, number of cases and controls per each study, array type, country, publication year, and DOI of reference.

GSE Accession	Tissue	Number of Cases	Number of Controls	Array Type	Country	Publication Year	Study DOI
GSE113725 [3]	Blood	48	48	Illumina 450K	UK	2018	10.1093/hmg/ddy199
GSE198904 [4]	Blood	548	100	Illumina 850K	USA	2022	10.1038/s41598-022-22744-6
GSE201287 [5]	Blood	40	40	Illumina 450K	China	2022	10.1073/pnas.2201967119
GSE125105 [6]	Blood	489	210	Illumina 450K	Germany	2019	10.1038/s41398-021-01756-2
GSE88890(BA2) [7]	Brain	17	18	Illumina 450K	UK	2017	10.1038/tp.2016.249
GSE88890(BA11) [7]	Brain	20	20	Illumina 450K	UK	2017	10.1038/tp.2016.249
GSE41826(Glia) [8]	Brain	29	29	Illumina 450K	USA	2013	10.4161/epi.23924
GSE41826(Neuron) [8]	Brain	29	29	Illumina 450K	USA	2013	10.4161/epi.23924

## Data Availability

The datasets presented in this study can be found in the online repository GEO (Gene Expression Omnibus), with the study accession numbers declared in Supplementary Table S1.

## Supplementary Materials

The following supporting information can be downloaded at: https://www.mdpi.com/article/10.3390/biomedicines12102181/s1, Table S1: Results of meta-analysis in whole blood tissue. Table S2: Results of meta-analysis in brain tissue. Table S3: Results of ORA analysis in whole blood. Table S4: Results of ORA analysis in brain. Table S5: Results of the Gene-EML analysis with the Fisher adjustment (blood and brain). Table S6: Results of ORA of the Gene-EML for KEGG pathways and Biological Process for whole blood tissue. Table S7: Results of ORA of the Gene-EML for KEGG pathways and Biological Process for brain tissue.
