# Supplementary material for: Investigating the Epigenetic Landscape of Major Depressive Disorder: A Genome-Wide Meta-Analysis of DNA Methylation Data, Including New Insights into Stochastic Epigenetic Mutations and Epivariations
Source: Biomedicines. 2024 Sep 25;12(10):2181. doi: 10.3390/biomedicines12102181 (PMC11505239; doi:10.3390/biomedicines12102181)
Supplement: Supplementary file 1 [file biomedicines-12-02181-s001.zip › Legend.pdf]

Supplementary Table S1: Results of meta-analysis in whole blood tissue

Supplementary Table S2: Results of meta-analysis in brain tissue

Supplementary Table S3: Results of ORA analysis in whole blood

Supplementary Table S4: Results of ORA analysis in brain

Supplementary Table S5: Results of the Gene-EML analysis with the Fisher adjustment (blood and brain)

Supplementary Table S6: Results of ORA of the Gene-EML for KEGG pathways and Biological Process for whole blood tissue

Supplementary Table S7: Results of ORA of the Gene-EML for KEGG pathways and Biological Process for brain tissue
