# Supplementary material for: Investigating the Epigenetic Landscape of Major Depressive Disorder: A Genome-Wide Meta-Analysis of DNA Methylation Data, Including New Insights into Stochastic Epigenetic Mutations and Epivariations
Source: Biomedicines. 2024 Sep 25;12(10):2181. doi: 10.3390/biomedicines12102181 (PMC11505239; doi:10.3390/biomedicines12102181)
Supplement: Supplementary file 1 [file biomedicines-12-02181-s001.zip › Supplementary Table S3.pdf]

**Enrichment Categories:** Geneontology_Biological_Process			
Gene Set	Description	P Value	**FDR**
GO:0009887	animal organ morphogenesis	<2.2e-16	<2.2e-16
GO:0010628	positive regulation of gene expression	9.9920e-16	3.7008e-12
GO:0022008	neurogenesis	1.2212e-15	3.7008e-12
GO:0051254	positive regulation of RNA metabolic process	2.6645e-15	6.0558e-12
GO:1902680	positive regulation of RNA biosynthetic process	5.5511e-15	9.6605e-12
GO:0010557	positive regulation of macromolecule biosynthetic process	6.8834e-15	9.6605e-12
GO:1903508	positive regulation of nucleic acid-templated transcription	7.4385e-15	9.6605e-12
GO:0031328	positive regulation of cellular biosynthetic process	9.5479e-15	1.0766e-11
GO:0009891	positive regulation of biosynthetic process	1.0658e-14	1.0766e-11
GO:0045893	positive regulation of transcription, DNA-templated	1.5099e-14	1.3727e-11

**Enrichment Categories:** pathway_KEGG			
Gene Set	Description	P Value	**FDR**
hsa04360	Axon guidance	9.0268e-7	0.00029427
hsa04510	Focal adhesion	6.1421e-06	0.0010012
hsa04722	Neurotrophin signaling pathway	6.7353e-05	0.0073191
hsa04934	Cushing syndrome	9.8121e-05	0.0076614
hsa05032	Morphine addiction	0.00011751	0.0076614
hsa04390	Hippo signaling pathway	0.00018894	0.0098896
hsa05200	Pathways in cancer	0.00021235	0.0098896

hsa04010	MAPK signaling pathway	0.00026321	0.010726
hsa04142	Lysosome	0.00039321	0.012859
hsa05226	Gastric cancer	0.00041843	0.012859

**Enrichment Categories:** Disease_Disgenet			
Gene Set	Description	P Value	**FDR**
C4020899	Autosomal recessive predisposition	7.0598e-12	2.5938e-8
C0036341	Schizophrenia	2.4030e-10	4.4142e-7
C0005586	Bipolar Disorder	2.7788e-8	0.000034031
C0025362	Mental Retardation	2.8498e-7	0.00012653
C0423903	Low intelligence	2.8498e-7	0.00012653
C0917816	Mental deficiency	2.8498e-7	0.00012653
C1843367	Poor school performance	2.8498e-7	0.00012653
C4020876	Dull intelligence	2.8498e-7	0.00012653
C3714756	Intellectual Disability	3.0995e-7	0.00012653
C0557874	Global developmental delay	7.6358e-7	0.00023378
