# Supplementary material for: Investigating the Epigenetic Landscape of Major Depressive Disorder: A Genome-Wide Meta-Analysis of DNA Methylation Data, Including New Insights into Stochastic Epigenetic Mutations and Epivariations
Source: Biomedicines. 2024 Sep 25;12(10):2181. doi: 10.3390/biomedicines12102181 (PMC11505239; doi:10.3390/biomedicines12102181)
Supplement: Supplementary file 1 [file biomedicines-12-02181-s001.zip › Supplementary Table S4.pdf]

**Enrichment Categories:** Geneontology_Biological_Process				
Gene Set	Description	Enrichment Ratio	P Value	**FDR**
GO:0006928	movement of cell or subcellular component	1.4285	<2.2e-16	<2.2e-16
GO:0040011	locomotion	1.4423	<2.2e-16	<2.2e-16
GO:0045595	regulation of cell differentiation	1.4722	<2.2e-16	<2.2e-16
GO:0007267	cell-cell signaling	1.4543	<2.2e-16	<2.2e-16
GO:0022008	neurogenesis	1.5523	<2.2e-16	<2.2e-16
GO:0030030	cell projection organization	1.5095	<2.2e-16	<2.2e-16
GO:0120036	plasma membrane bounded cell projection organization	1.5181	<2.2e-16	<2.2e-16
GO:0022603	regulation of anatomical structure morphogenesis	1.5836	<2.2e-16	<2.2e-16
GO:0000902	cell morphogenesis	1.6663	<2.2e-16	<2.2e-16
GO:0000904	cell morphogenesis involved in differentiation	1.7207	<2.2e-16	<2.2e-16

**Enrichment Categories:** pathway_KEGG				
Gene Set	Description	Enrichment Ratio	P Value	**FDR**
hsa04510	Focal adhesion	1.6940	4.8394e-06	0.0015776
hsa04512	ECM-receptor interaction	2.0555	1.7589e-05	0.0022945
hsa04360	Axon guidance	1.6855	2.2134e-05	0.0022945
hsa04151	PI3K-Akt signaling pathway	1.4582	2.8153e-05	0.0022945
hsa04072	Phospholipase D signaling pathway	1.6956	8.4242e-05	0.0054926
hsa05165	Human papillomavirus infection	1.4139	0.00018305	0.0081812
hsa04933	AGE-RAGE signaling pathway in diabetic complications	1.8090	0.00019993	0.0081812

hsa05200	Pathways in cancer	1.3218	0.00020076	0.0081812
hsa04928	Parathyroid hormone synthesis, secretion and action	1.7392	0.00038358	0.013894
hsa05145	Toxoplasmosis	1.6781	0.00068441	0.022312

**Enrichment Categories:** Disease_Disgenet				
Gene Set	Description	Enrichment Ratio	P Value	**FDR**
C0036341	Schizophrenia	1.4276	1.1673e-11	4.2888e-8
C0005586	Bipolar Disorder	1.4767	2.5699e-7	0.00047209
C0024421	Macroglossia	1.6855	2.2134e-05	0.0022945
C0023893	Liver Cirrhosis, Experimental	1.3457	2.4526e-06	0.0022528
C4020899	Autosomal recessive predisposition	1.2342	3.3795e-06	0.0023253
C0004352	Autistic Disorder	1.5872	3.7974e-06	0.0023253
C0009806	Constipation	1.8389	5.717e-06	0.0030006
C0237326	Dyschezia	1.8273	1.0686e-05	0.0049078
C0376634	Craniofacial Abnormalities	1.7537	2.4551e-05	0.0099518
C0019322	Umbilical hernia	2.0785	2.7087e-05	0.0099518
